# Superficial Inferior Epigastric Artery Flap for a Total Parotidectomy Defect

**Published:** 2017-12-18

**Authors:** Lee W. T. Alkureishi, Mihir K. Bhayani, Mark Sisco

**Affiliations:** ^a^Plastic Surgery, Shriners Hospitals for Children, Chicago, Ill; ^b^Divisions of Otolaryngology; ^c^Plastic Surgery, Northshore University Healthsystem, Evanston, Ill

**Keywords:** free flap, parotidectomy, facial asymmetry, microsurgery

## Abstract

**Introduction:** The superficial inferior epigastric artery flap offers ample volume for reconstruction, an inconspicuous scar, and no functional donor site deficit. This report details its use for volume replacement after parotidectomy. **Methods:** We report a 27-year-old woman with recurrent acinic cell carcinoma, requiring left total parotidectomy and partial mastoidectomy. In anticipation of significant contour deficit and postoperative radiation, reconstruction with a superficial inferior epigastric artery adipose-free flap was performed. **Results:** Resection and reconstruction were carried out with no complications. The postoperative course was uneventful, with recovery of facial nerve function and an aesthetic, symmetrical outcome. The donor site scar is completely hidden by underwear. **Conclusion:** The superficial inferior epigastric artery flap represents an underused option in head and neck reconstruction. It offers similar benefits to that of the parascapular flap but with the advantages of a 2-team approach and a less conspicuous donor scar.

With continued improvements in anatomical knowledge and microsurgical techniques, the variety of options for head and neck reconstruction is greater than ever before. The choice of reconstructive technique demands a delicate balance between form, function, and donor site morbidity.

In patients with facial volume loss in the absence of a skin defect, the options for volume replacement include free dermal fat graft, lipoaspirate fat transfer, and vascularized free tissue transfer. Nonvascularized fat transfer remains a reasonable choice in selected patients; however, the results of this are generally unpredictable for larger volume defects or in the setting of radiotherapy,^1^ where survival of the transferred fat volume is significantly impaired. Lipoaspirate fat transfer has found use for many facial volume defects; however, its utility is limited by inability to place the fat at the time of the resection, unpredictable fat survival, and the need for multiple treatments. In addition, in the setting of delayed reconstruction for parotidectomy volume deficits, the facial nerve lies in close proximity to the surface skin and is vulnerable to injury from repeated insertions of the cannula.

Conversely, free flap techniques provide a means of immediate reconstruction with well-vascularized fatty tissue and reliable, consistent survival of the transferred volume. In addition, vascularized fat is more resistant to the effects of subsequent radiation, helping mitigate any secondary deformity.^2^

The choice of donor site for free flap transfer is dependent on multiple factors and must be tailored to the defect, reconstructive requirements, body habitus, and ultimately the patient's wishes. For many patients, the parascapular flap satisfies most of these criteria and has become a favored option for many surgeons.^3^^,^^4^ However, the superficial inferior epigastric artery (SIEA) flap provides another option, with a number of potential advantages over the parascapular flap. To date, there are very few reports detailing its use in the face,^5^^,^^6^ and this is the first report describing an SIEA flap reconstruction following a total parotidectomy.

## METHODS

**Figure 1:**
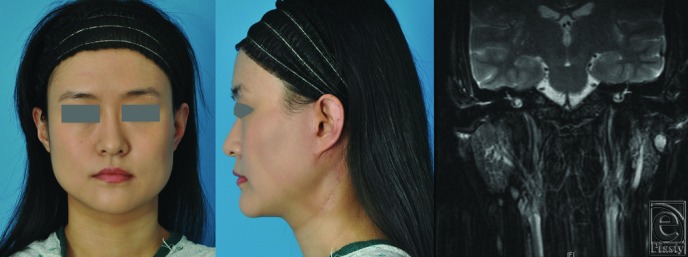
Preoperative presentation: (a) frontal view and (b) lateral view. (c) Magnetic resonance image demonstrating tumor within the left parotid gland.

A review of the patient's chart and preoperative/postoperative imaging was carried out and is presented here. A 27-year-old otherwise healthy Asian American woman presented with recurrent acinic cell carcinoma of the left parotid gland. Her original tumor was staged T1N0 and was treated with superficial parotidectomy at another institution in 2012. In February 2015, the patient noted a mass in the preauricular area. Examination revealed a slim young woman with a body mass index of 19. She had intact facial nerve function, a preauricular scar from her prior resection, and a palpable subcutaneous nodule anterior to the scar (Figs 1*a* and 1*b*). MRI demonstrated multiple nodules within the substance of the parotid (Fig 1*c*), and an ultrasound-guided fine needle aspirate confirmed the diagnosis of recurrent acinic cell carcinoma.

The multidisciplinary head and neck team recommended she undergo total parotidectomy with facial nerve preservation. In view of the anticipated contour defect, exposed facial nerve, and likelihood of requiring postoperative radiation, reconstruction with a free vascularized adipose flap transfer was recommended.

At operation, total parotidectomy was performed with identification and preservation of all facial nerve branches, facilitated by partial mastoidectomy with decompression of the proximal facial nerve trunk. The resection specimen weighed 15 g.

Concurrently, a pencil Doppler probe was used to identify signals from the SIEA and superficial inferior epigastric vein (SIEV) in the right groin, over which a 4-cm transverse incision was made. Through this incision, the vessels were identified and dissected under loupe magnification. The caliber and quality were felt to be adequate to support the flap and so the incision was extended to complete the dissection back to the vessels’ origins from the external iliac artery and vein.

**Figure 2:**
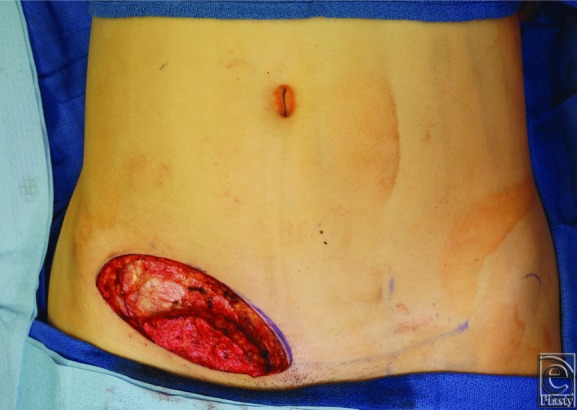
Intraoperative view demonstrating the superficial inferior epigastric artery flap, which has been de-epithelialized in situ prior to transfer.

The resection specimen was used to guide the dimensions of the flap, which was de-epithelialized in situ (Fig 2). The flap was rendered ischemic and transferred to the face for microvascular anastomosis to the superficial temporal vessels. A 2.0-mm coupler (Synovis Micro, Birmingham, Ala) was used for the venous anastomosis, whereas the 1.3-mm arterial anastomosis was completed with interrupted 10-0 nylon sutures. Satisfactory perfusion was confirmed, and the flap was inset into the defect before closing the preauricular incision. The donor site was closed in layered fashion, with undermining of the cephalic abdominal skin.

## RESULTS

**Figure 3:**
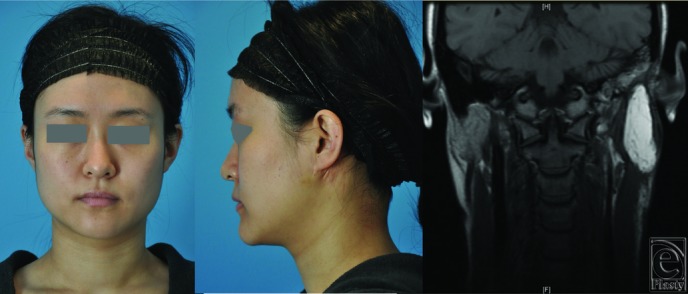
Postoperative images: (a) frontal view and (b) lateral view. Excellent facial symmetry is evident. (c) Postoperative magnetic resonance image demonstrating the superficial inferior epigastric artery flap within the parotidectomy bed.

**Figure 4:**
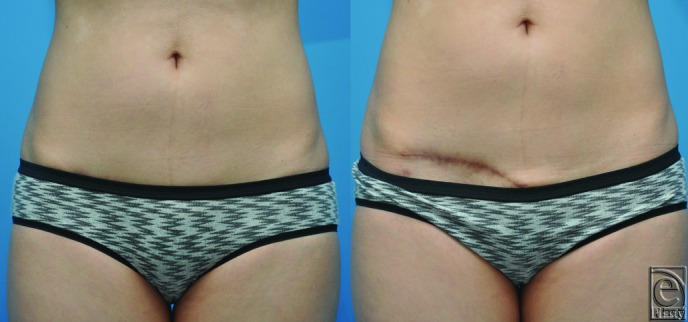
Donor site. Postoperative photographs demonstrating a favorable donor site scar. (a) Hidden by underwear and (b) with underwear lowered. There is some residual color in the scar, which is expected to improve further with time.

Her postoperative course was unremarkable, and she was discharged on postoperative day 2. She experienced transient facial nerve weakness that fully recovered after 2 months. She completed adjuvant proton therapy. Nine months after surgery, and 5 months after completion of radiation therapy, facial volume symmetry was excellent (Figs 3*a*-3*c*), although she did have mild residual skin hyperpigmentation from radiation in the preauricular area. At the donor site, the scar was completely hidden by underwear (Fig 4). The patient remains disease-free.

## DISCUSSION

The parascapular flap has found popularity as the workhorse solution for free flap reconstruction in patients with facial volume loss, particularly in those without associated skin deficits. It is widely accepted as the first choice for volume restoration in patients with Parry-Romberg disease^3^ and is increasingly finding use following tumor ablation.^4^ The advantages of the parascapular flap include reliable vascular anatomy, ease of dissection, and the availability of multiple tissue types. However, flap harvest may require an intraoperative position change and the resultant scar is located in a relatively conspicuous area.

The SIEA flap utilizes tissue from the lower abdomen, a widely used donor site for breast reconstruction.^7^ The advantages of this donor site include ample volume of available fat, a large available skin paddle, an inconspicuous scar, and the potential added benefit of an abdominoplasty-type procedure. In this very slim patient, the donor site yielded a supple flap with plentiful volume for the reconstruction and without a resultant abdominal contour deformity. In addition, dissection of the superficial inferior epigastric vessels is performed without disruption of the abdominal wall fascia, avoiding the potential for abdominal wall weakness or bulge that may result from transverse rectus abdominis myocutaneous or deep inferior epigastric perforator flap harvest. The SIEA flap is also harvested in the supine position, avoiding an intraoperative position change and facilitating a 2-team approach. In our opinion, these characteristics favor the use of the SIEA flap over the parascapular flap in patients with volume asymmetry of the face.

Despite finding favor for use in breast reconstruction and elsewhere, the SIEA flap remains an infrequently utilized option in the head and neck surgery, with only 2 case reports describing its use in the setting of hemifacial microsomia or traumatic contour defects.^5^^,^^6^ The flap was also described as part of a combined soft tissue and bony reconstruction with the deep circumflex iliac artery flap in a series of 12 patients with oral cancer in 2011.^8^

The main disadvantage of the SIEA flap relates to inconsistent vascular anatomy, with previous reports suggesting that the vessel is absent in one third of patients and of insufficient caliber in another one third.^9^ However, the majority of studies evaluating this flap have been in the breast reconstruction literature, where the vessels are required to support very large tissue volumes. In the setting of facial volume loss, the required flap volume is significantly less and a smaller caliber vessel will often be sufficient.

The SIEA vessel may still be completely absent in up to one third of patients, in which case secondary flap options are necessary. The superficial circumflex artery and vein can often be found slightly more lateral within the same donor site and may represent a secondary option in these patients.^10^ Alternatively, other adipocutaneous or adipose tissue flap donor sites such as the parascapular flap, thoracodorsal artery perforator (TAP) flap, or anterolateral thigh flap can be good tertiary options. In the current patient, a pencil Doppler probe was used to locate the course of the SIEA and SIEV vessels prior to incision, and a limited, 4-cm incision was initially made to evaluate vessel caliber before committing to a larger scar. In addition, preoperative discussion with the patient included the possibility of switching to the TAP flap donor site in the event that no useable vessels were found.

## CONCLUSION

The free SIEA flap is an underused and potentially useful option for reconstruction of total parotidectomy defects. This report is the first to describe several important advantages of this donor site over the more commonly used parascapular flap in this application.
